# Study on Storage Stability of Activated Reclaimed Rubber Powder Modified Asphalt

**DOI:** 10.3390/ma14164684

**Published:** 2021-08-19

**Authors:** Peipei Kong, Gang Xu, Jingyao Yang, Xianhua Chen, Yaqin Zhu

**Affiliations:** 1 School of Transportation, Southeast University, Nanjing 211189, China; xc_kong@seu.edu.cn (P.K.); xugang619@hotmail.com (G.X.); yangjingyao19@foxmail.com (J.Y.); 2 Jiangsu Zhonghong Environment Technology Co., Ltd., Jiangyin 214434, China; Zhuyq@163.com

**Keywords:** lubricant by-products, reclaimed rubber powder, modified asphalt, storage stability, microstructure

## Abstract

The purpose of this research was to make full use of waste lubricating by-products (LBP) and reclaimed rubber powder (RR) to modify asphalt by a one-pot approach, so as to achieve the dual purpose of solving the poor storage stability of reclaimed rubber powder modified asphalt (RRMA) and the realization of solid waste recycling. A variety of characterization techniques were performed to analyze storage stability, conventional properties and microstructure of LBP-activated reclaimed rubber powder modified asphalt (Blend). Fourier transform infrared spectroscopy illustrated that not only the chemical composition of LBP was very similar to that of asphalt, but also the activation of LBP improved the compatibility of RR with asphalt and enhanced the storage stability of Blend. Fluorescence spectrum and scanning electron microscopy results indicated that the RR without LBP activation was aggregated and dispersed as blocks in asphalt, while the LBP activated RR was uniformly dispersed in the asphalt phase. The segregation test demonstrated that Blend exhibited outstanding storage stability, in which the softening point difference was within 2.5 °C and the segregation rate was −0.2–0.2. In addition, the conventional properties of Blend have been significantly improved, especially in penetration and ductility. More importantly, the short-term aging results demonstrated that, compared with RRMA, Blend possessed excellent anti-aging performance.

## 1. Introduction

Asphalt has been widely used due to its good plasticity, cohesiveness and viscoelasticity [1,2]. However, it has exposed some defects such as easily cracking at low temperature and easily softening and flow at high temperature, which greatly limit the scope of its application [3,4]. In order to further promote the serviceability performance of asphalt, researchers have put forward a variety of improvement measures through unremitting efforts [5,6,7,8,9]. Among the many measures, the modification of asphalt using waste rubber powder was the most promising. It has been reported that waste rubber powder modified asphalt can not only improve the high temperature permanent deformation resistance, low temperature crack resistance, and can inhibit thermal oxygen aging, but also can improve the comfort and safety of vehicle driving, extend the service life of the pavement, and reduce traffic noise [10,11,12,13,14]. More importantly, the application of waste rubber in modified asphalt can not only realize the resource utilization of waste rubber but also reduce the hazards to the environment [15,16].

However, the application of universal waste rubber with an integral three-dimensional network structure to modified asphalt not only increased the viscosity of modified asphalt, but also caused the separation of the rubber phase from the asphalt phase [17,18,19,20]. Surprisingly, reclaimed rubber powder, prepared by destroying the molecular cross-linking structure of ordinary rubber powder, modified asphalt, which effectively solved the problems of high viscosity, inferior compatibility and difficult construction of universal rubber powder modified asphalt [21,22,23,24]. The reason for this was that under the action of high temperature and high shear force, the surface of reclaimed rubber powder produces some active groups, which is conducive to the chemical bonding with asphalt, improving the storage stability of the modified asphalt [25,26,27,28]. For example, Wang et al. [26] evaluated the performance of devulcanized rubber asphalt based on rheology and environmental effects. It was found that devulcanized rubber asphalt has better storage stability and low temperature performance than vulcanized rubber asphalt. Li et al. [27] studied the physics, rheology and stability of devulcanized rubber asphalt and rubber powder asphalt and found that devulcanized rubber asphalt and rubber powder asphalt have similar physical and rheological properties, but devulcanized rubber asphalt was much better in improving storage performance. The main reason was that devulcanized rubber can integrate into asphalt to perform both chemical reaction and physical swelling while crumb rubber mainly dispersed incompatibly in asphalt. However, segregation still emerged after long-term high temperature storage [29]. The lubrication by-product (LBP) with affluent active aromatic components was a low value-added product of the lubricant production process, which was no longer suitable as raw materials of fluid catalytic cracking units or else coking units and therefore generally used as fuels [30]. It not only fritters resources but also contaminating the environment. The research literature revealed that the chemical composition of LBP was similar to asphalt, and according to the similar solubility theory, LBP could be used as excellent pre-swelling materials [31]. The rubber powder was not only activated but also its structure was expanded under the soaking and swelling of LBP [32]. To the best of our knowledge, we found that the published researches only reported the swelling effect of LBP on waste rubber powder, and there was no exhaustive information on the storage stability and microstructure of LBP-activated reclaimed rubber modified asphalt.

The objective of this work was to investigate the effect of LBP on the conventional properties, storage stability and aging performance of reclaimed rubber powder modified asphalt, as well as to explore the microstructure of LBP pretreated rubber powder modified asphalt using advanced characterization. Finally, a novel method for preparing reclaimed rubber powder modified asphalt with high storage stability was explored.

## 2. Materials and Methods

### 2.1. Materials

The lubricant by-product (LBP) used in this paper was from the lubricant manufactured (PetroChina Lanzhou Petrochemical Company, Lanzhou, China), and its properties were shown in Table 1. The asphalt in this paper was the 70# matrix asphalt, the penetration grade of which was 70. The 40 mesh (425 μm) reclaimed rubber powder and the stabilizer were supplied by specialized manufacturers.

### 2.2. Preparation of the Blend

**Figure 1:**
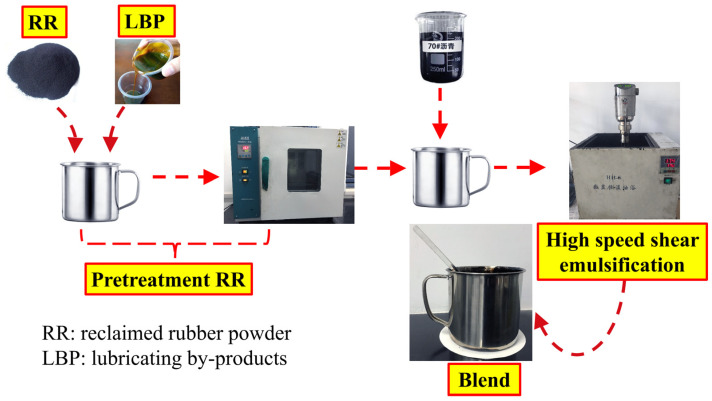
The preparation route of Blend.

The Blend was prepared by the one-pot method and the flow was as shown in Figure 1. The lubricant by-products and reclaimed rubber powder were mixed in a certain ratio, and the mixture was activated in the oven at about 150 °C for 1 h. Then the preheated 70# asphalt was added to the mixture and was emulsified by high-speed shear at 180 °C and stirred at a rate of 5000 r/min for 1 h. Finally, the stabilizer was added and electric stirring (3000 r/min) was carried out at 180 °C for 1 h to prepare the Blend.

### 2.3. Measurement and Characterization

#### 2.3.1. Fourier Transform Infrared Spectroscopy (FTIR)

In order to investigate the effects of LBP, reclaimed rubber powder on the functional groups and chemical compositions of modified asphalt, the FTIR spectrometer (Nicolet iS10, Thermo Nicolet Corp., Waltham, MA, USA) was used to determine the information of the chemical structure of LBP, reclaimed rubber powder modified asphalt RRMA and Blend. The wavenumber range of the test was from 400 to 4000 cm^−1^.

#### 2.3.2. Scanning Electron Microscopy (SEM)

The morphology of the RRMA and Blend were observed by scanning electron microscopy (SEM, S-4300, Hitachi Co., Ltd., Tokyo, Japan).

#### 2.3.3. Fluorescence Microscopy

The state of reclaimed rubber powder modified asphalt and Blend were observed by fluorescence microscopy. A small molten sample was first loaded between two glass slides, which were then squashed carefully. Then, three groups of thin glass slide with sample were observed at room temperature under optical microscopy of Olympus IX71 (Tokyo, Japan).

#### 2.3.4. Conventional Properties

The conventional properties, such as ductility, softening point, penetration, and viscosity were tested according to ASTM specifications [33,34,35,36]. The aging properties were conducted using a thin film oven test (TFOT) aging at 163 °C ± 1 °C for 5 h according to ASTM specifications [37]. For each conventional property reported, at least three samples measurement were averaged.

#### 2.3.5. Dynamic Shear Rheometer (DSR) Tests

Dynamic rheological properties (such as phase angle, complex modulus, and so on) of 70# asphalt, RRMA and Blend were obtained from DSR (MCR, Anton Paar Company, Graz, Austria). The DSR tests temperature was 25 °C and the angular frequency was 10 rad/s. For each DSR reported, at least three samples measurement were averaged.

### 2.4. Segregation Tests

The modified asphalt was heated evenly to avoid local overheating, while stirring, injected into the sample container of the tester. Setting the storage temperature to 163 °C, leave the oven to stand for 48 ± 1 h without any disturbance, the separation test tube with the bracket was gently removed from the oven and put into the low-temperature test chamber, keeping the separation test tube in an upright position, so that the modified asphalt specimens were solidified, then the separation test tube was removed from the test chamber after all curing. When the temperature of the sample was slightly softened, the sample tube was cut into three equal sections with scissors, and the top 1/3 and bottom 1/3 of the sample were sampled, and the softening point test and DSR were conducted. For the segregation tests, at least three samples measurement were averaged.

## 3. Results and Discussion

### 3.1. FTIR Analysis

**Figure 2:**
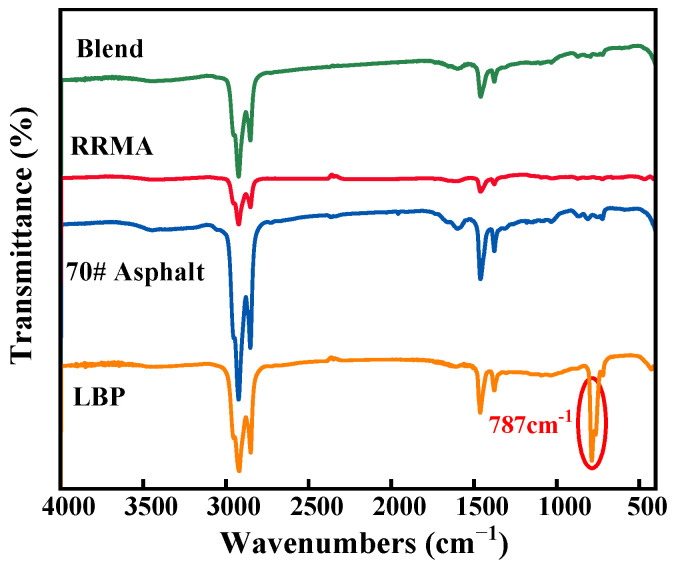
FTIR spectrum of LBP, 70# asphalt, RRMA and Blend.

The FTIR spectra of LBP, 70# asphalt, RRMA and Blend were presented in Figure 2. It can be observed from Figure 2 that the characteristic peaks of LBP, 70# asphalt, RRMA and Blend were almost the identical (2926 and 2852 cm^−1^: C–H aliphatic stretching; 1599 cm^−1^: the aromatic C=C stretching; 1459 cm^−1^: C-H aliphatic index bending in CH_2_; 1374 cm^−1^: the C–H aliphatic branched bending in CH_3_), and the only distinction was the different intensity of the characteristic peaks and the aromatic groups appearing at 787 cm^−1^ for LBP. Combined with the results in Table 1, it could be determined that LBP was mainly composed of aromatic compounds [30]. It was well known that aromatic compounds were an essential component of asphalt and was the dispersion medium for gum soluble asphaltenes. Therefore, the loss of aromatic components in the asphalt (e.g., swelling, oxidation) not only changed the colloidal structure of the asphalt, but also severely weakened the low-temperature performance of the modified asphalt. However, the FTIR spectra showed that LBP and asphalt possessed similar aromatic components. This fact indicated that LBP can effectively compensate for the loss of aromatic components in 70# asphalt [32]. It can also be shown that LBP can improve the compatibility of reclaimed rubber powder and asphalt, thereby improving the storage stability of modified asphalt.

### 3.2. Microstructure Characterization

#### 3.2.1. SEM Analysis

**Figure 3:**
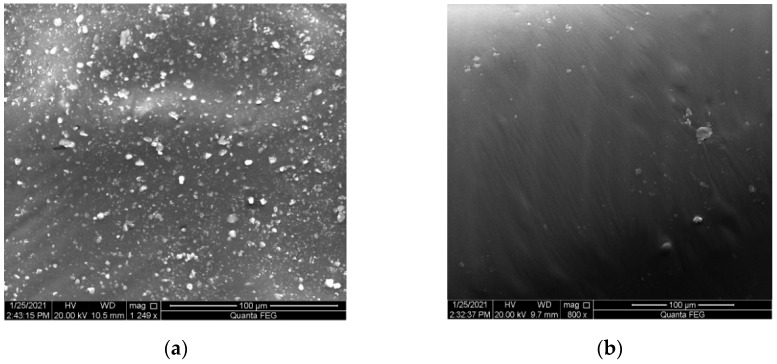
SEM of (**a**) RRMA and (**b**) Blend.

In order to more clearly and intuitively observe the dispersion state of reclaimed rubber powder in asphalt, scanning electron microscopy (SEM) tests were conducted on the RRMA and Blend, the results were shown in Figure 3. It can be observed from Figure 3a that the surface of RRMA was uneven and possessed an obvious granular structure. The reason for this phenomenon was the low surface energy of reclaimed rubber powder, not easy to occur with the asphalt to fully swell the combination, which ultimately led to different sizes and shapes of reclaimed rubber powder particles dispersed in the asphalt [14,27]. On the contrary, the LBP-activated reclaimed rubber powder can be better dispersed in the asphalt, and there was no obvious formation of large particles. The reason for this interesting phenomenon was that the activation of LBP not only improved the surface energy of the reclaimed rubber powder, but also fully swelled the sulfur bridge structure of the reclaimed rubber powder. Therefore, the activation of LBP was beneficial to improve the compatibility of reclaimed rubber powder and asphalt, so that the reclaimed rubber powder particles were more evenly dispersed in the asphalt.

#### 3.2.2. Fluorescence Microscopy Analysis

Fluorescence microscopy is an extremely important tool for the study of microstructures and microscopic changes. Accordingly, the composition and location of fluorescence substances in the sample could be observed and distinguished using fluorescence. As is well known, in polymer modified asphalt, when the polymer swelling in the asphalt phase is irradiated by short wavelength light, it can excite a longer wavelength fluorescence, but the asphalt phase did not emit light. More importantly, the light of this wavelength cannot damage the real morphological structure of the polymer phase in modified asphalt. Therefore, the microscopic morphology of the RRMA and Blend could be observed with fluorescence microscopy.

**Figure 4:**
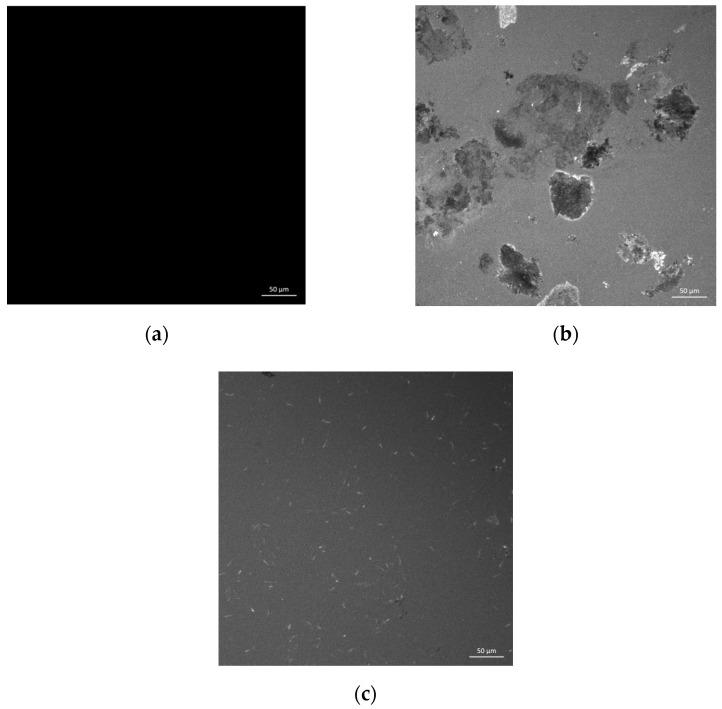
The fluorescence spectrum of (**a**) 70# asphalt; (**b**) RRMA; (**c**) Blend.

In this study, the fluorescence spectrum of 70# asphalt, RRMA and Blend were shown in Figure 4. As can be seen in Figure 4a, the fluorescence spectrum of 70# asphalt under fluorescence microscopy presented black, mainly because the asphalt phase did not emit light under fluorescence. As can be seen from Figure 4b, the reclaimed rubber powder particles without LBP activation were aggregated and dispersed as blocks in asphalt. At the same time, an obvious interface was observed between the rubber and asphalt, indicating that their compatibility was poor. From Figure 4c, it can be found that the LBP activated reclaimed rubber powder was not agglomerated, and the rubber particles were evenly dispersed in the asphalt. The main reason was that LBP weakened the interaction between the molecular chains of the reclaimed rubber powder, swelling the dense sulfur bridge structure and softening the reclaimed rubber powder sufficiently, so that the reclaimed rubber powder was evenly dispersed in the asphalt [23]. Therefore, fluorescence microscopy analysis further proved that the activation of LBP improved the storage stability and compatibility of the modified asphalt.

### 3.3. Storage Stability

When the RRMA was stored at high temperature, as the density of reclaimed rubber powder was higher than the asphalt, the reclaimed rubber powder uniformly distributed in the asphalt phase was prone to sedimentation and segregation under the action of gravity. The segregation rate (as shown in Equation (1)) was used as an indicator to evaluate the storage stability of modified asphalt [28].

*S*_r_ = [(*G*_b_*/sin*δ*_b_)/(*G*_t_*/sin*δ*_t_)] − 1 (1)

where *S*_r_ is segregation rate, *G*_t_*, *G*_b_* are the upper and lower complex modulus, respectively. The*δ*_t_,*δ*_b_ are the upper and lower phase angle, respectively. *G*_t_*/sin*δ*_t_, *G*_b_*/sin*δ*_b_ are the rutting factor of the upper and lower samples under the storage conditions of 163 °C and 48 ± 1 h.

**Figure 5:**
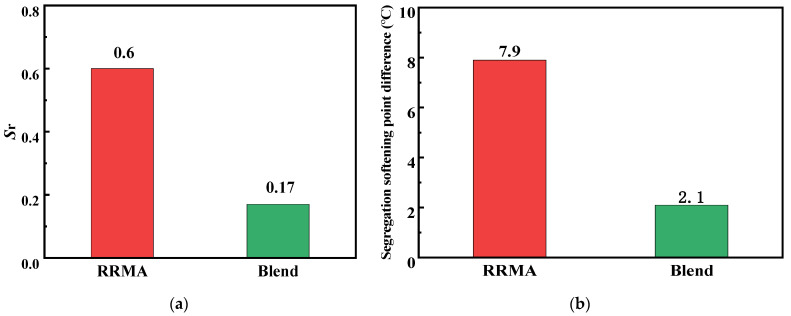
The (**a**) segregation rate (Sr); (**b**) segregation softening point difference of RRMA and Blend.

The storage stability test results of RRMA and Blend were shown in Table 2. The relationship between the segregation rate (*S*_r_) and softening point difference were shown in Figure 5.

According to the standard of polymer modified asphalt, when the softening point difference between the upper and lower 1/3 segments is less than 2.5 °C, the product is considered qualified. In addition, the storage stability of modified asphalt can also be evaluated based on the segregation rate (*S*_r_). When −0.2 ≤ *S*_r_ ≤ 0.2, the modified asphalt storage stability was qualified; on the contrary, it was considered unqualified. As can be seen from Table 2 and Figure 5, the LBP played an important role in improving storage stability. By adding a certain amount of LBP, the segregation softening point difference of modified asphalt could be significantly reduced, and its value was within 2.5 °C. More importantly, the *S*_r_ of modified asphalt was between −0.2–0.2, and the conclusions obtained from the two indicators were basically the same, which indicated that LBP-activated the reclaimed rubber powder modified asphalt possessed excellent storage stability. This phenomenon can be attributed to the fact that the LBP containing active aromatic components not only sufficiently swelled the reclaimed rubber powder, but also made the density of the LBP activated reclaimed rubber powder closer to the density of the base asphalt. As a result, the storage stability of LBP activated reclaimed rubber powder modified asphalt was improved [23,25,28].

### 3.4. Conventional Properties

It can be seen from Table 3 that the conventional performance of LBP-activated reclaimed rubber powder modified asphalt was obviously improved compared with the 70# asphalt and RRMA. Among them, the penetration of RRMA and Blend at 25 °C was reduced compared to that of 70# asphalt, while the 5 °C ductility, softening point, and 135 °C viscosity were increased compared to that of 70# asphalt. This indicated that the temperature sensitivity of RRMA and Blend was reduced, the high temperature performance was improved, and the low temperature ductility was increased. In addition, it can also be found that the 25 °C penetration and 5 °C ductility of Blend were significantly higher than those of RRMA. This indicated that the activation effect of LBP improved the low temperature performance of Blend, and to some extent weakened the high temperature performance of Blend [20]. It was also worth noting that the 135 °C viscosity of Blend was lower than of RRMA. The reason for this phenomenon may be attributed to the fact that LBP increased the proportion of aromatic components in the asphalt and weakened the asphalt-rubber interaction, thus reduced the rotational viscosity of Blend [28].

### 3.5. Anti-Aging Performance

Aging is the key factor affecting the service life of the asphalt pavement. In order to quantitatively evaluate the influence of LBP on the thermal oxidative aging resistance of Blend, this work measured the softening point, penetration and ductility of RRMA and Blend after TFOT test. Subsequently, the corresponding aging indexes including residual penetration ratio (*K*_p_, as shown in Equation (2)) and softening point increment (Δ*T*, as shown in Equation (3)), were calculated to measure the degree of modified asphalt aging.

*K*_p_ = (*P*_2_/*P*_1_) × 100% (2)

where *K*_p_ is residual penetration ratio (%), *P*_1_ is the penetration of the original sample before aging (0.1 mm), *P*_2_ is the penetration of the original sample after aging (0.1 mm).

Δ*T* = *T*_2_ − *T*_1_ (3)

where Δ*T*, *T*_1_, *T*_2_ are softening point difference (°C), softening point before aging (°C) and softening point after aging (°C), respectively.

**Figure 6:**
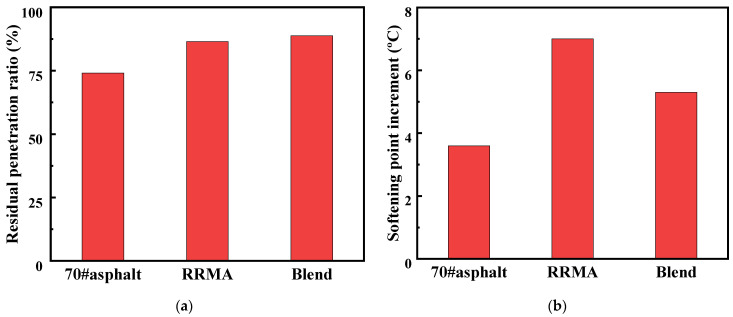
Aging indices of 70# asphalt, RRMA and Blend after TFOT aging: (**a**) residual penetration ratio; (**b**) softening point increment.

As shown in Figure 6, the penetration and ductility of RRMA and Blend decreased after TFOT aging, while the corresponding softening point values of the two asphalt binders increased after aging, and the increase of RRMA was greater than that of Blend. This experimental phenomenon was consistent with the fact that short-term aging led to the hardening of asphalt binders. At the same time, the susceptibility of 70# asphalt, RRMA and Blend to TFOT aging effects were evaluated using the aging indexes *K*_p_ and Δ*T*. The smaller value of *K*_p_ and the larger value of Δ*T* indicated that the corresponding asphalt binder was more susceptible to aging and less resistant to aging. As shown in Figure 6, the Δ*T* of Blend was less than RRMA and 70# asphalt. This indicated that the addition of LBP enhanced the anti-aging property of Blend [28,32].

## 4. Conclusions

In this paper, modified asphalt with excellent storage properties was successfully prepared by a one-pot method using waste LBP and reclaimed rubber powder. Based on the obtained results, the following conclusions were drawn based on experiments:

1. The Fourier infrared spectroscopy demonstrated that the LBP and 70# asphalt have similar chemical components. It indicated that LBP can improve the compatibility of reclaimed rubber powder and asphalt.
2. Fluorescence spectra and SEM revealed that the LBP activated reclaimed rubber powder can be better dispersed in asphalt, and there was no obvious agglomerated. Therefore, the activation of LBP was beneficial to improve the compatibility of reclaimed rubber powder and asphalt.
3. The segregation test indicated that the LBP activated reclaimed rubber powder modified asphalt possessed excellent storage stability, in which the softening point difference was within 2.5 °C and the segregation rate was −0.2–0.2.
4. Compared with 70# asphalt and RRMA, the conventional properties of LBP activated reclaimed rubber powder modified asphalt has been significantly improved. The activation effect of LBP improved the low temperature performance of Blend, and reduced the rotational viscosity Blend.
5. The TFOT results manifested that the anti-aging performance of LBP activated reclaimed rubber powder modified asphalt was superior to that of RRMA.

## Figures and Tables

**Table 1:** Physical properties of lubricant by-products.

Items	Measured Values
Flash point (°C)	≥200
Kinematic viscosity (100 °C, mm^2^/s)	24
Aniline point (°C)	<32
Specific gravity (20 °C)	1.01
Aromatics content (%)	80
Moisture content (%)	<0.1
Sulfur content (%)	<0.08

**Table 2:** DSR test results of RRMA and Blend.

Asphalt Binder	*G*_t_*/kPa	*G*_b_*/kPa	*δ*_t_	*δ*_b_	*S*_r_	*T*_t_/°C	*T*_b_/°C	ΔT/°C
RRMA	1834	2634	54.4	48.7	0.60	64.35	72.25	7.90
Blend	2485	3088	44.3	52.2	0.17	77.35	79.45	2.10

**Table 3:** Conventional properties of 70# asphalt, RRMA and Blend.

Items	70# Asphalt	RRMA	Blend
Penetration (25 °C, 0.1 mm)	60.6	50.3	58.8
Softening point (°C)	54.7	61.6	60.1
Ductility (5 °C, 5 cm/min, cm)	>100	10.1	11.3
135 °C viscosity (Pa·s)	0.49	7.2	6.1
Segregation softening point difference (°C)	-	7.9	2.1
**Residue after TFOT**			
Residual penetration ratio (25 °C), %	74.1	86.5	88.8
Ductility ratio (5 °C), %	-	72.6	76.8
Softening point increment (°C)	3.6	7.0	5.3

## Data Availability

Data is contained within the article.
